# Soft Translations and Soft Extensions of BCI/BCK-Algebras

**DOI:** 10.1155/2014/536709

**Published:** 2014-09-14

**Authors:** Nazra Sultana, Nazia Rani, Muhammad Irfan Ali, Azhar Hussain

**Affiliations:** ^1^Department of Mathematics, University of Sargodha, Sargodha 40100, Pakistan; ^2^Department of Mathematics, University of Sargodha, Women Campus, Faisalabad, Pakistan; ^3^Islamabad Model College for Girls F-6/2, Islamabad 44000, Pakistan

## Abstract

The concept of soft translations of soft subalgebras and soft ideals over BCI/BCK-algebras is introduced and some related properties are studied. Notions of Soft extensions of soft subalgebras and soft ideals over BCI/BCK-algebras are also initiated. Relationships between soft translations and soft extensions are explored.

## 1. Introduction

Recently soft set theory has emerged as a new mathematical tool to deal with uncertainty. Due to its applications in various fields of study researchers and practitioners are showing keen interest in it. As enough number of parameters is available here, so it is free from the difficulties associated with other contemporary theories dealing with uncertainty. Prior to soft set theory, probability theory, fuzzy set theory, rough set theory, and interval mathematics were common mathematical tools for dealing with uncertainties, but all these theories have their own difficulties. These difficulties may be due to lack of parametrization tools [1, 2]. To overcome these difficulties, Molodtsov [2] introduced the concept of soft sets. A detailed overview of these difficulties can be seen in [1, 2]. As a new mathematical tool for dealing with uncertainties, Molodtsov has pointed out several directions for the applications of soft sets. Theoretical development of soft sets is due to contributions from many researchers. However in this regard initial work is done by Maji et al. in [1]. Later Ali et al. [3] introduced several new operations in soft set theory.

At present, work on the soft set theory is progressing rapidly. Maji et al. [4] described the application of soft set theory in decision making problems. Aktaş and Çağman studied the concept of soft groups and derived their basic properties [5]. Chen et al. [6] proposed parametrization reduction of soft sets, and then Kong et al. [7] presented the normal parametrization reduction of soft sets. Feng and his colleagues studied roughness in soft sets [8, 9]. Relationship between soft sets, fuzzy sets, and rough sets is investigated in [10]. Park et al. [11] worked on notions of soft WS-algebras, soft subalgebras, and soft deductive system. Jun and Park [12] presented the notions of soft ideals, idealistic soft, and idealistic soft BCI/BCK-algebras. Further applications of soft sets can be seen in [13–25].

The study of BCI/BCK-algebras was initiated by Imai and Iseki [26] as the generalization of concept of set theoretic difference and propositional calculus. For the general development of BCI/BCK-algebras, the ideal theory and its fuzzification play an important role. Jun et al. [27–30] studied fuzzy trends of several notions in BCI/BCK-algebras. Application of soft sets in BCI/BCK is given in [12, 31].

Translations play a vital role in reducing the complexity of a problem. In geometry it is a common practice to translate a system to some new position to study its properties. In linear algebra translations help find solution to many practical problems. In this paper idea of translations is being extended to soft BCI/BCK algebras.

This paper is arranged as follows: in Section 2, some basic notions about BCI/BCK-algebra and soft sets are given. These notions are required in the later sections. Concept of translation is introduced in Section 3 and some properties of it are discussed here.  Section 4 is devoted for the study of soft ideal translation in BCI/BCK-algera. In Section 5, concept of ideal extension is introduced and some of its properties are studied.

## 2. Preliminaries

First of all some basic concepts about BCI/BCK-algebra are given. For a comprehensive study on BCI/BCK-algebras [32] is a very nice monograph by Meng and Jun. Then some notions about soft sets are presented here as well.

An algebra (*X*, ∗, 0) is called a BCI-algebra if it satisfies the following conditions:

1. (∀*x*, *y*, *z* ∈ *X*)  (((*x*∗*y*)∗(*x*∗*z*))∗(*z*∗*y*) = 0),
2. (∀*x*, *y* ∈ *X*)  ((*x*∗(*x*∗*y*))∗*y* = 0),
3. (∀*x* ∈ *X*)  (*x*∗*x* = 0),
4. (∀*x*, *y* ∈ *X*)  (*x*∗*y* = 0, *y*∗*x* = 0⇒*x* = *y*).

If a BCI-algebra satisfies the following identity:

(5) (∀*x* ∈ *X*)  (0∗*x* = 0),

then *X* is called a BCK-algebra. Any BCK-algebra satisfies the following axioms:

- (∀*x* ∈ *X*)  (*x*∗0 = *x*),
- (∀*x*, *y*, *z* ∈ *X*)  (*x*∗*y* = 0⇒(*x*∗*z*)∗(*y*∗*z*) = 0, (*z*∗*y*)∗(*z*∗*x*) = 0),
- (∀*x*, *y*, *z* ∈ *X*)  ((*x*∗*y*)∗*z* = (*x*∗*z*)∗*y*),
- (∀*x*, *y*, *z* ∈ *X*)  (((*x*∗*z*)∗(*y*∗*z*))∗(*x*∗*y*) = 0).

A subset *S* of a BCI/BCK-algebra *X* is called a subalgebra of *X* if *x*∗*y* ∈ *S*, for all *x*, *y* ∈ *S*.

A subset *A* of a BCI/BCK-algebra *X* is called an ideal of *X*, denoted by *A*⊲*X*, if it satisfies:

1. 0 ∈ *A*,
2. (∀*x*, *y* ∈ *X*)  (*x*∗*y* ∈ *A*, *y* ∈ *A*⇒*x* ∈ *A*).

Now we recall some basic notions in soft set theory. Let *U* be a universe and *E* be a set of parameters. Let *P*(*U*) denote the power set of *U* and let *A*, *B* be nonempty subsets of *E*.

**Definition 1 (see [2]).**

A pair (*F*, *A*) is called a soft set over *U*, where *F* is a mapping given by *F* : *A* → *P*(*U*).

**Definition 2 (see [3]).**

Let *U* be a universe, let *E* be the set of parameters, and let *A*⊆*E*.

- (*F*, *A*) is called a relative null soft set (with respect to the parameters set *A*), denoted by *∅*_*A*_, if *F*(*a*) = *∅*, for all *a* ∈ *A*.
- (*G*, *A*) is called a relative whole soft set (with respect to the parameters set *A*), denoted by *U*_*A*_, if *G*(*e*) = *U*, for all *e* ∈ *A*.

**Definition 3 (see [3]).**

The complement of a soft set (*F*, *A*) is denoted by (*F*, *A*)^*c*^ and is defined by (*F*, *A*)^*c*^ = (*F* ^*c*^, *A*), where *F* ^*c*^ : *A* → *P*(*U*) is a mapping given by *F* ^*c*^(*a*) = *U* − *F*(*a*), ∀*a* ∈ *A*. Clearly, ((*F*, *A*)^*c*^)^*c*^ = (*F*, *A*).

**Definition 4 (see [8]).**

A soft set (*F*, *A*) over *U* is called a full soft set if ⋃_*a*∈*A*_ *F*(*a*) = *U*.

## 3. Soft Translations of Soft Subalgebras

Here notion of translations in soft BCI/BCK-algebra is initiated. Concept of soft extensions is introduced here also.

Let *F*_*A*_ : *X* → *P*(*X*) be set valued map defined as

(1) $$\begin{array}{c} F_{A}\left(x\right)\neq \emptyset \;\text{if}\;\;x\in A, \end{array}$$

where *A*⊆*X*. Then *F*_*A*_ also denotes a soft set over a BCI/BCK algebra *X*. From here onward a soft set will be denoted by symbols like *F*_*A*_, unless stated otherwise.

A soft set *F*_*A*_ over a BCI/BCK-algebra *X* is called a soft subalgebra of *X* if it satisfies

(2) $$\begin{array}{c} \left(\forall x,y\in X\right)\;\left(F_{A}\left(x*y\right)⊇F_{A}\left(x\right)\cap F_{A}\left(y\right)\right). \end{array}$$

In what follows *X* = (*X*, ∗, 0) denote a BCI/BCK-algebra, and for any soft set *F*_*A*_ over *X*, we denote *T* : = *X* − ∪{*F*_*A*_(*x*)∣*x* ∈ **X**} unless otherwise specified.

That is *T* = (⋃_*x*∈*X*_ *F*_*A*_(*x*))^*c*^ = ⋂_*x*∈*X*_ *F*_*A*_^*c*^(*x*).

It is easy to see that *T*∩*F*_*A*_(*x*) = *∅* for all *x* ∈ *X*. If *F*_*A*_ is a full soft set then *T* is an empty set. Therefore throughout this paper only those soft set are considered which are not full.

**Definition 5 .**

Let *F*_*A*_ be a soft set over *X* and let *U*_1_⊆*T*. A mapping *F*_*U*_1__^*T*^ : *X* → *P*(*X*) is called a soft *U*_1_-translation of *F*_*A*_ if, for all *x* ∈ *X*,

(3) $$\begin{array}{c} F_{U_{1}}^{T}\left(x\right)=F_{A}\left(x\right)\cup U_{1}. \end{array}$$

**Lemma 6 .**

Let *U*_1_⊆*T* and *F*_*A*_ be a soft set over *X*, then *F*_*A*_(*x*) ∪ *U*_1_⊇*F*_*A*_(*y*) ∪ *U*_1_ implies *F*_*A*_(*x*)⊇*F*_*A*_(*y*), for all *x*, *y* ∈ *X*.

**Proof**

Since *U*_1_⊆*T*, *F*_*A*_(*x*)∩*U*_1_ = *∅* and *F*_*A*_(*y*)∩*U*_1_ = *∅*. Let *a* ∈ *F*_*A*_(*y*) then *a* ∈ *F*_*A*_(*y*) ∪ *U*_1_⊆*F*_*A*_(*x*) ∪ *U*_1_ this implies *a* ∈ *F*_*A*_(*x*) or *a* ∈ *U*_1_ but *a* ∉ *U*_1_ because *F*_*A*_(*y*)∩*U*_1_ = *∅*. So *a* ∈ *F*_*A*_(*x*) that is *F*_*A*_(*x*)⊇*F*_*A*_(*y*), for all *x*, *y* ∈ *X*.

**Proposition 7 .**

Let *F*_*A*_ be a soft subalgebra of *X* and *U*_1_⊆*T*. Then the soft *U*_1_-translation *F*_*U*_1__^*T*^ of *F*_*A*_ is a soft subalgebra of *X*.

**Proof**

Let *x*, *y* ∈ *X*. Then

(4) FU1T(x∗y)=FA(x∗y)∪U1⊇(FA(x)∩FA(y))∪U1=(FA(x)∪U1)∩(FA(y)∪U1)=(FU1T(x))∩(FU1T(y)).

Hence *F*_*U*_1__^*T*^ is a soft subalgebra of *X*.

**Proposition 8 .**

Let *F*_*A*_ be a soft set over *X* such that the *U*_1_-translation *F*_*U*_1__^*T*^ of *F*_*A*_ is a soft subalgebra of *X* for some *U*_1_⊆*T*. Then *F*_*A*_ is a soft subalgebra of *X*.

**Proof**

Assume *F*_*U*_1__^*T*^ is a soft subalgebra of *X* for some *U*_1_⊆*T*. Let *x*, *y* ∈ *X*, we have

(5) FA(x∗y)∪U1=FU1T(x∗y)⊇FU1T(x)∩FU1T(y)=(FA(x)∪U1)∩(FA(y)∪U1)=(FA(x)∩(y))∪U1.

Now by Lemma 6 we have

(6) $$\begin{array}{c} F_{A}\left(x*y\right)⊇F_{A}\left(x\right)\cap F_{A}\left(y\right), \end{array}$$

for all *x*, *y* ∈ *X*. Hence *F*_*A*_ is a soft subalgebra of *X*.

From Propositions 7 and 8 we have the following.

**Theorem 9 .**

A soft set *F*_*A*_ of *X* is a soft subalgebra of *X* if and only if *U*_1_-translation *F*_*U*_1__^*T*^ of *F*_*A*_ is a soft subalgebra of *X* for some *U*_1_⊆*T*.

**Definition 10 .**

Let *F*_*A*_ and *G*_*B*_ be two soft sets over *X*. If *F*_*A*_(*x*)⊆*G*_*B*_(*x*) for all *x* ∈ *X*, then we say that *G*_*B*_ is a soft extension of *F*_*A*_.

**Example 11 .**

Consider a BCI/BCK-algebra *X* = {0,1, 2,3} presented as follows:

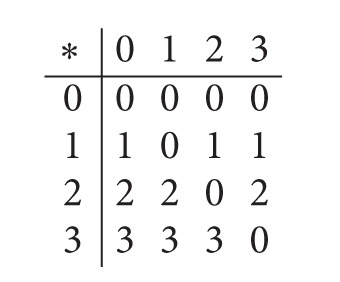
(7)
Define two soft sets *F*_*A*_ and *G*_*B*_ of *X* as in Table 1.

Here *F*_*A*_(0)⊆*G*_*B*_(0), *F*_*A*_(1)⊆*G*_*B*_(1), *F*_*A*_(2)⊆*G*_*B*_(2), and *F*_*A*_(3)⊆*G*_*B*_(3), which implies that *G*_*B*_ is a soft extension of *F*_*A*_.

Next the concept of soft *S*-extension is being introduced.

**Definition 12 .**

Let *F*_*A*_ and *G*_*B*_ be two soft sets over *X*. Then *G*_*B*_ is called a soft *S*-extension of *F*_*A*_, if the following conditions hold:

1. *G*_*B*_ is a soft extension of *F*_*A*_.
2. If *F*_*A*_ is a soft subalgebra of *X*, then *G*_*B*_ is a soft subalgebra of *X*.

As we know *F*_*U*_1__^*T*^(*x*)⊇*F*_*A*_(*x*) for all *x* ∈ *X*. As a consequence of Definition 12 and Theorem 9, we have the following.

**Theorem 13 .**

Let *F*_*A*_ be a soft subalgebra of *X* and *U*_1_⊆**T**. Then the soft *U*_1_-translation *F*_*U*_1__^*T*^ of *F*_*A*_ is a soft *S*-extension of *F*_*A*_.

The converse of Theorem 13 is not true in general as seen in the following example.

**Example 14 .**

Consider a BCI/BCK-algebra *X* = {0,1, 2,3} given as follows:

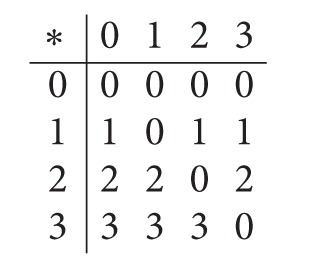
(8)
Define a soft set *F*_*A*_ of *X* by Table 2.

Then *F*_*A*_ is a soft subalgebra of *X*. For soft set *F*_*A*_, *T* = {3}. Let *G*_*B*_ be a soft set over *X* given by Table 3.

Then *G*_*B*_ is a soft *S*-extension of *X*. But it is not a soft *U*_1_-translation of *F*_*A*_ for any nonempty *U*_1_⊆*T*.

For a soft set *F*_*A*_ of *X*, *U*_1_⊆*T* and *U*_2_ ∈ *P*(*X*) with *U*_2_⊇*U*_1_, let

(9) $$\begin{array}{c} U_{U_{1}}\left(F_{A};U_{2}\right):=\left\{x\in X\;|\;F_{A}\left(x\right)⊇U_{2}-U_{1}\right\}. \end{array}$$

If *F*_*A*_ is a soft subalgebra of *X*, then it is clear that *U*_*U*_1__(*F*_*A*_; *U*_2_) is a subalgebra of *X* for all *U*_2_ ∈ *P*(*X*) with *U*_2_⊇*U*_1_. But, if we do not give condition that *F*_*A*_ is a soft subalgebra of *X*, then *U*_*U*_1__(*F*_*A*_; *U*_2_) may not be a subalgebra of *X* as seen in the following example.

**Example 15 .**

Let *X* = {0,1, 2,3, 4} be a BCI/BCK-algebra presented as follows:

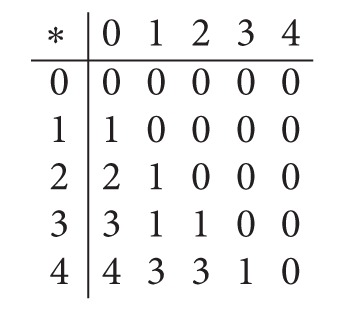
(10)

Define a soft subset *F*_*A*_ of *X* by Table 4.

Then *F*_*A*_ is not a soft subalgebra of *X* with *T* = {1}. Since *F*_*A*_(3∗4) = {0}⊉{0,4} = *F*_*A*_(3)∩*F*_*A*_(4) For *U*_2_ = {1,4} and *U*_1_ = {1}, we obtain *U*_*U*_1__(*F*_*A*_; *U*_2_) = {3,4} which is not a subalgebra of *X* since 3∗3 = 0 ∉ *U*_*U*_1__(*F*_*A*_; *U*_2_).

In the following theorem, relationship between *U*_1_-translations and *U*_*U*_1__(*F*_*A*_; *U*_2_) is studied in case of soft subalgebra.

**Theorem 16 .**

Let *F*_*A*_ be a soft set over *X* and *U*_1_⊆*T*. Then the soft *U*_1_-translation *F*_*U*_1__^*T*^ of *F*_*A*_ is a soft subalgebra of *X* if and only if *U*_*U*_1__(*F*_*A*_; *U*_2_) is a subalgebra of *X* for all *U*_2_ ∈ *P*(*U*) with *U*_2_⊇*U*_1_.

**Proof**

Assume that the soft *U*_1_-translation *F*_*U*_1__^*T*^ of *F*_*A*_ is a soft subalgebra of *X*. Then by Theorem 9, *F*_*A*_ is a soft subalgebra of *X* if *F*_*U*_1__^*T*^ is a soft subalgebra of *X*. Further let *a*, *b* ∈ *U*_*U*_1__(*F*_*A*_; *U*_2_), then *F*_*A*_(*a*)⊇*U*_2_ − *U*_1_ and *F*_*A*_(*b*)⊇*U*_2_ − *U*_1_ are subalgebras of *X* for all *U*_2_ ∈ *P*(*U*) with *U*_2_⊇*U*_1_. Consider

(11) $$\begin{array}{c} F_{A}\left(a*b\right)⊇F_{A}\left(a\right)\cap F_{A}\left(b\right)⊇U_{2}-U_{1}. \end{array}$$

Therefore *a*∗*b* ∈ *U*_*U*_1__(*F*_*A*_; *U*_2_), which shows that *U*_*U*_1__(*F*_*A*_; *U*_2_) is a subalgebra of *X*, for all *U*_2_⊆*P*(*U*), with *U*_2_⊇*U*_1_.

Conversely, suppose that *U*_*U*_1__(*F*_*A*_; *U*_2_) is a subalgebra of *X* for all *U*_2_⊆*P*(*U*) with *U*_2_⊇*U*_1_. Now assume that there exist *a*, *b* ∈ *X* such that

(12) $$\begin{array}{c} F_{U_{1}}^{T}\left(a*b\right)\subset U_{2}\subseteq F_{U_{1}}^{T}\left(a\right)\cap F_{U_{1}}^{T}\left(b\right). \end{array}$$

Then *F*_*A*_(*a*)⊇*U*_2_ − *U*_1_ and *F*_*A*_(*b*)⊇*U*_2_ − *U*_1_ but *F*_*A*_(*a*∗*b*) ⊂ *U*_2_ − *U*_1_. This shows that *a*, *b* ∈ *U*_*U*_1__(*F*_*A*_; *U*_2_) and *a*∗*b* ∉ *U*_*U*_1__(*F*_*A*_; *U*_2_), which is a contradiction and so *F*_*U*_1__^*T*^(*a*∗*b*)⊇*F*_*U*_1__^*T*^(*a*)∩*F*_*U*_1__^*T*^(*b*) for all *a*, *b* ∈ *X*. Hence *F*_*U*_1__^*T*^ is a soft subalgebra of *X*.

**Theorem 17 .**

Let *F*_*A*_ be a soft subalgebra of *X* and let *U*_1_, *U*_2_⊆*T*. If *U*_1_⊇*U*_2_, then the soft *U*_1_-translation *F*_*U*_1__^*T*^ of *F*_*A*_ is a soft *S*-extension of the soft *U*_2_-translation *F*_*U*_2__^*T*^ of *F*_*A*_.

**Proof**

Since *U*_1_⊇*U*_2_, this implies *F*_*U*_1__^*T*^(*x*)⊇*F*_*U*_2__^*T*^(*x*), for all *x* ∈ *X*. So *U*_1_-translation is an extension of *U*_2_-translation, and from Theorem 9, *F*_*U*_1__^*T*^ and *F*_*U*_2__^*T*^ are soft subalgebras of *F*_*A*_. Hence soft *U*_1_-translation *F*_*U*_1__^*T*^ of *F*_*A*_ is a soft *S*-extension of the soft *U*_2_-translation *F*_*U*_2__^*T*^ of *F*_*A*_.

For every soft subalgebra *F*_*A*_ of *X* and *U*_2_⊆*T*, the soft *U*_2_-translation *F*_*U*_2__^*T*^ of *F*_*A*_ is a soft subalgebra of *X*. If *G*_*B*_ is a soft *S*-extension of *F*_*U*_2__^*T*^ and then there exists *U*_1_⊆*T* such that *U*_1_⊇*U*_2_ and *G*_*B*_(*x*)⊇*F*_*U*_1__^*T*^(*x*), for all *x* ∈ *X*. Thus, we have the following theorem.

**Theorem 18 .**

Let *F*_*A*_ be a soft subalgebra of *X* and *U*_2_⊆*T*. For every soft *S*-extension *G*_*B*_ of soft *U*_2_-translation *F*_*U*_2__^*T*^ of *F*_*A*_, there exists a *U*_1_⊆*T* such that *U*_1_⊇*U*_2_ and *G*_*B*_ are a soft *S*-extension of *U*_1_-translation of *F*_*A*_.

**Proof**

For every soft subalgebra *F*_*A*_ of *X* and *U*_2_⊆*T*, the soft *U*_2_-translation *F*_*U*_2__^*T*^ of *F*_*A*_ is a soft subalgebra of *X*. If *G*_*B*_ is a soft *S*-extension of *F*_*U*_2__^*T*^ and then there exists *U*_1_⊆*T* such that *U*_1_⊇*U*_2_ and *G*_*B*_(*x*)⊇*F*_*U*_1__^*T*^(*x*), for all *x* ∈ *X*. Then by Theorem 17, *G*_*B*_ is a soft *S*-extension of *U*_1_-translation of *F*_*A*_.

**Definition 19 .**

A soft *S*-extension *G*_*B*_ of a soft subalgebra *F*_*A*_ of *X* is said to be normalized if there exists *x*_0_ ∈ *X* such that *G*_*B*_(*x*_0_) = *X*.

**Definition 20 .**

Let *F*_*A*_ be a soft subalgebra of *X*. A soft set *G*_*B*_ of *X* is called a maximal soft *S*-extension of *F*_*A*_ if it satisfies the following conditions:

1. *G*_*B*_ is a soft *S*-extension of *F*_*A*_,
2. there does not exist another soft subalgebra of *X* which is a soft extension of *G*_*B*_.

**Example 21 (see [33]).**

Let *Z* ^+^ be a set of positive integers and let “∗” be a binary operation on *Z* ^+^ defined by

(13) $$\begin{array}{c} x*y=\frac{x}{\left(x,y\right)}, \end{array}$$

∀*x*, *y* ∈ *Z* ^+^, where (*x*, *y*) is the greatest common divisor of *x* and *y*. Then (*Z* ^+^; ∗, 1) is a BCK-algebra. Let *F*_*A*_ and *G*_*B*_ be soft sets of *Z* ^+^ which are defined by *F*_*A*_(*x*) = {1,2, 3} and *G*_*B*_(*x*) = *Z* ^+^ for all *x* ∈ *Z* ^+^. Clearly, *F*_*A*_ and *G*_*B*_ are soft subalgebras of *Z* ^+^. By using definition of maximal soft *S*-extension, then it is easy to see that *G*_*B*_ is a maximal soft *S*-extension of *F*_*A*_.

**Proposition 22 .**

If a soft set *G*_*B*_ of *X* is a normalized soft *S*-extension of a soft subalgebra *F*_*A*_ of *X*, then *G*_*B*_(0) = *X*.

**Proof**

Assume that *G*_*B*_ is a normalized soft *S*-extension of a soft subalgebra *F*_*A*_ of *X* then there exists *x*_0_ ∈ *X* such that *G*_*B*_(*x*_0_) = *X*, for some *x*_0_ ∈ *X*. Consider

(14) $$\begin{array}{c} G_{B}\left(0\right)=G_{B}\left(x_{0}*x_{0}\right)⊇G_{B}\left(x_{0}\right)\cap G_{B}\left(x_{0}\right)=X. \end{array}$$

This implies *G*_*B*_(0) = *X*.

**Theorem 23 .**

Let *F*_*A*_ be a soft subalgebra of *X*. Then every maximal soft *S*-extension of *F*_*A*_ is normalized.

**Proof**

This follows from the definitions of the maximal and normalized soft *S*-extensions.

## 4. Soft Translations of Soft Ideals in Soft BCI/BCK-Algebras

Now concept of translation of a soft ideal of a BCI/BCK-algebra is introduced.

**Definition 24 .**

A soft subset *F*_*A*_ of a BCI/BCK-algebra is called a soft ideal of *X*, denoted by *F*_*A*_⊲_*S*_ *X*, if it satisfies:

1. (∀*x* ∈ *X*) (*F*_*A*_(0)⊇*F*_*A*_(*x*)),
2. (∀*x*, *y* ∈ *X*) (*F*(*x*)⊇(*F*_*A*_(*x*∗*y*)∩*F*_*A*_(*y*))).

**Theorem 25 .**

If *F*_*A*_ is a soft subset of *X*, then *F*_*A*_ is a soft ideal of *X* if and only if soft *U*_1_-translation *F*_*U*_1__^*T*^ of *F*_*A*_ is a soft ideal of *X* for all *U*_1_⊆*T*.

**Proof**

Assume that *F*_*A*_⊲_*S*_ *X* and let *U*_1_⊆*T*. Then *F*_*U*_1__^*T*^(0) = *F*_*A*_(0) ∪ *U*_1_⊇*F*_*A*_(*x*) ∪ *U*_1_ = *F*_*U*_1__^*T*^(*x*) and

(15) FU1T(x)=FA(x)∪U1⊇(FA(x∗y)∩FA(y))∪U1=(FA(x∗y)∪U1)∩(FA(y)∪U1)=FU1T(x∗y)∩FU1T(y) ∀x,y∈X.            Hence  FU1T⊲SX.

Conversely, assume that *F*_*U*_1__^*T*^ is a soft ideal of *X* for some *U*_1_⊆*T*. Let *x*, *y* ∈ *X*. Then

(16) FU1T(0)⊇FU1T(x)⟹FA(0)∪U1⊇FA(x)∪U1⟹FA(0)⊇FA(x)  by  Lemma  6,

and so *F*_*A*_(0)⊇*F*_*A*_(*x*). Next

(17) FA(x)∪U1=FU1T(x)⊇FU1T(x∗y)∩FU1T(y)=(FA(x∗y)∪U1)∩(FA(y)∪U1)=(FA(x∗y)∩FA(y))∪U1,

which implies that *F*_*A*_(*x*)⊇*F*_*A*_(*x*∗*y*)∩*F*_*A*_(*y*) (by Lemma 6). Hence *F*_*A*_ is a soft ideal of *X*.

## 5. Soft Extensions and Soft Ideal Extensions of Soft Subalgebras

In this section concept of soft ideal extension is being introduced and some of its properties are studied.

**Definition 26 .**

Let *F*_*A*_ and *G*_*B*_ be the soft subsets of *X*. Then *G*_*B*_ is called the soft ideal extension of *F*_*A*_, if the following conditions hold:

1. *G*_*B*_ is a soft extension of *F*_*A*_.
2. *F*_*A*_⊲_*S*_ *X*⇒*G*_*B*_⊲_*S*_ *X*.

For a soft subset *F*_*A*_ of *X*, *U*_1_⊆*T* and *U*_2_ ∈ *P*(*X*) with *U*_2_⊇*U*_1_, define *E*_*U*_1__(*F*_*A*_; *U*_2_): = {*x* ∈ *X*∣*F*_*A*_(*x*) ∪ *U*_1_⊇*U*_2_}.

It is clear that if *F*_*A*_⊲_*S*_ *X*, then *U*_*U*_1__(*F*_*A*_; *U*_2_)⊲*X* for all *U*_2_ ∈ *P*(*U*) with *U*_2_⊇*U*_1_.

**Theorem 27 .**

For *U*_1_⊆*T*, let *F*_*U*_1__^*T*^ be the soft *U*_1_-translation of *F*_*A*_. Then the following are equivalent:

1. *F*_*U*_1__^*T*^⊲_*S*_ *X*.
2. (∀*U*_2_ ∈ *P*(*U*)) (*U*_2_⊃*U*_1_⇒*E*_*U*_1__(*F*_*A*_; *U*_2_)⊲*X*).

**Proof**

(1)⇒(2) Consider *F*_*U*_1__^*T*^⊲_*S*_ *X* and let *U*_2_ ∈ *P*(*U*) be such that *U*_2_⊃*U*_1_. Since *F*_*U*_1__^*T*^(0)⊇*F*_*U*_1__^*T*^(*x*) for all *x* ∈ *X*, we have

(18) $$\begin{array}{c} F_{A}\left(0\right)\cup U_{1}=F_{U_{1}}^{T}\left(0\right)⊇F_{U_{1}}^{T}\left(x\right)=F_{A}\left(x\right)\cup U_{1}⊇U_{2}, \end{array}$$

for *x* ∈ *E*_*U*_1__(*F*_*A*_; *U*_2_).

(19) $$\begin{array}{c} \text{Hence}\;\;0\in E_{U_{1}}\left(F_{A};U_{2}\right). \end{array}$$

Let *x*, *y* ∈ *X* be such that *x*∗*y* ∈ *E*_*U*_1__(*F*_*A*_; *U*_2_) and *y* ∈ *E*_*U*_1__(*F*_*A*_; *U*_2_). Then *F*_*A*_(*x*∗*y*) ∪ *U*_1_⊇*U*_2_ and *F*_*A*_(*y*) ∪ *U*_1_⊇*U*_2_, that is, *F*_*U*_1__^*T*^(*x*∗*y*) = *F*_*A*_(*x*∗*y*) ∪ *U*_1_⊇*U*_2_ and *F*_*U*_1__^*T*^(*y*) = *F*_*A*_(*y*) ∪ *U*_1_⊇*U*_2_. Since *F*_*U*_1__^*T*^⊲_*S*_ *X*, it follows that

(20) $$\begin{array}{c} F_{A}\left(x\right)\cup U_{1}=F_{U_{1}}^{T}\left(x\right)⊇F_{U_{1}}^{T}\left(x*y\right)\cap F_{U_{1}}^{T}\left(y\right)⊇U_{2}, \end{array}$$

that is, *F*_*A*_(*x*) ∪ *U*_1_⊇*U*_2_ so that *x* ∈ *E*_*U*_1__(*F*_*A*_; *U*_2_). Therefore *E*_*U*_1__(*F*_*A*_; *U*_2_)⊲*X*.

(2)⇒(1) Suppose that *E*_*U*_1__(*F*_*A*_; *U*_2_)⊲*X* for every *U*_2_ ∈ *P*(*U*) with *U*_2_⊇*U*_1_. If there exists *x* ∈ *X* with *U*_3_⊇*U*_1_ such that *F*_*U*_1__^*T*^(0) ⊂ *U*_3_⊆*F*_*U*_1__^*T*^(*x*) and then *F*_*A*_(*x*) ∪ *U*_1_⊇*U*_3_ but *F*_*A*_(0) ∪ *U*_1_ ⊂ *U*_3_. This shows that *x* ∈ *E*_*U*_1__(*F*_*A*_; *U*_2_) and 0 ∉ *E*_*U*_1__(*F*_*A*_; *U*_2_). This is a contradiction, and so *F*_*U*_1__^*T*^(0)⊇*F*_*U*_1__^*T*^(*x*), for all *x* ∈ *X*.

Now assume that there exist *a*, *b* ∈ *X* such that *F*_*U*_1__^*T*^(*a*) ⊂ *U*_4_⊆*F*_*U*_1__^*T*^(*a*∗*b*)∩*F*_*U*_1__^*T*^(*b*). Then *F*_*A*_(*a*∗*b*) ∪ *U*_1_⊇*U*_4_ and *F*_*A*_(*b*) ∪ *U*_1_⊇*U*_4_, but *F*_*A*_(*a*) ∪ *U*_1_ ⊂ *U*_4_. Hence *a*∗*b* ∈ *E*_*U*_1__(*F*_*A*_; *U*_4_) and *b* ∈ *E*_*U*_1__(*F*_*A*_; *U*_4_), but *a* ∉ *E*_*U*_1__(*F*_*A*_; *U*_4_). This is impossible and therefore *F*_*U*_1__^*T*^(*x*)⊇*F*_*U*_1__^*T*^(*x*∗*y*)∩*F*_*U*_1__^*T*^(*y*), for all *x*, *y* ∈ *X*. Consequently *F*_*U*_1__^*T*^⊲_*S*_ *X*.

**Theorem 28 .**

Let *F*_*A*_⊲_*S*_ *X* and *U*_1_, *U*_2_⊆*T*. If  *U*_1_⊇*U*_2_, then the soft *U*_1_-translation *F*_*U*_1__^*T*^ of *F*_*A*_ is a soft ideal extension of the soft *U*_2_-translation *F*_*U*_2__^*T*^ of *F*_*A*_.

**Proof**

Since

(21) $$\begin{array}{c} F_{U_{1}}^{T}\left(x\right)=F_{A}\left(x\right)\cup U_{1},\;\;F_{U_{2}}^{T}\left(x\right)=F_{A}\left(x\right)\cup U_{2}, \end{array}$$

*U*_1_⊇*U*_2_, this implies that (*F*_*U*_1__^*T*^(*x*)⊇*F*_*U*_2__^*T*^(*x*))  (∀*x* ∈ *X*). This shows that *F*_*U*_1__^*T*^ is a soft extension of *F*_*U*_2__^*T*^.

Now, let *F*_*U*_2__^*T*^ is a soft ideal of *X*, then *F*_*U*_1__^*T*^(0) = *F*_*A*_(0) ∪ *U*_1_⊇*F*_*A*_(*x*) ∪ *U*_1_ = *F*_*U*_1__^*T*^(*x*) for all *x* ∈ *X*, so we have (*F*_*U*_1__^*T*^(0)⊇*F*_*U*_1__^*T*^(*x*)). Consider

(22) FU1T(x)=FA(x)∪U1⊇(FA(x∗y)∩FA(y))∪U1=(FA(x∗y)∪U1)∩(FA(y)∪U1)=FU1T(x∗y)∩FU1T(y)  for all x,y∈X.

That is (*F*_*U*_1__^*T*^(*x*)⊇*F*_*U*_1__^*T*^(*x*∗*y*)∩*F*_*U*_1__^*T*^(*y*))  (∀*x*, *y* ∈ *X*) so *F*_*U*_1__^*T*^ is a soft ideal of *X*. Hence *F*_*U*_1__^*T*^ is a soft ideal extension of *F*_*U*_2__^*T*^.

## 6. Conclusion

Soft set theory is a mathematical tool to deal with uncertainties. Translation and extension are very useful concepts in mathematics to reduce the complexity of a problem. These concepts are frequently employed in geometry and algebra. In this papers, we presented some new notions such as soft translations and soft extensions for BCI/BCK-algebras. We also examined some relationships between soft translations and soft extensions. Moreover, soft ideal extensions and translations have been introduced and investigated as well. It is hoped that these results may be helpful in other soft structures as well.

## Figures and Tables

**Table 1:** 

*X*	0	1	2	3
*F*_*A*_	{0}	{0,1}	{0,2}	{1,2}
*G*_*B*_	{0}	{0,1, 2}	{0,2}	{0,1, 2}

**Table 2:** 

*X*	0	1	2	3
*F*_*A*_	{0,1, 2}	{0,1}	{0,2}	{1,2}

**Table 3:** 

*X*	0	1	2	3
*G*_*B*_	{0,1, 2}	{0,1, 2}	{0,2}	{0,1, 2}

**Table 4:** 

*X*	0	1	2	3	4
*F*_*A*_	{0}	{0,2}	{0,2, 3}	{0,3, 4}	{0,4}
